# Trimerization of CD40L-specific affibody molecules using collagen domains enhances target binding and CD40 blockade

**DOI:** 10.1007/s00018-026-06301-2

**Published:** 2026-06-24

**Authors:** Cornelia Westerberg, Chiara Sorini, Mariam Al-Haddad, Hanna Mehari, Charlotta Preger, Stefan Ståhl, Maja Jagodic, John Löfblom

**Affiliations:** 1 Department of Protein Science, School of Engineering Sciences in Chemistry, Biotechnology and Health, KTH Royal Institute of Technology, Stockholm, 106 91 Sweden; 2 Department of Clinical Neuroscience, Center for Molecular Medicine, Karolinska Institutet, Karolinska University Hospital, Stockholm, 171 76 Sweden

**Keywords:** Affibody, CD40L, CD40, Trimerization domain, Collagen XV, Collagen XVIII

## Abstract

Due to its central role in regulating adaptive immune responses through CD40 engagement, the CD40 ligand (CD40L) represents an attractive therapeutic target for inflammatory and autoimmune diseases. Here, CD40L-specific affibody molecules were generated by directed evolution using an *Escherichia coli* surface display platform. Two affibody candidates were identified that specifically recognized human CD40L on the surface of activated primary T cells. To exploit the homotrimeric architecture of CD40L, the affibody candidates were genetically fused to the NC1 domains of human collagen XV or XVIII, yielding well-defined homotrimeric constructs. The trimeric affibody formats were characterized with respect to oligomeric state, thermal stability, binding kinetics, cellular target engagement, and functional inhibition of CD40 signaling. NC1-mediated trimerization resulted in an approximately 100-fold improvement in apparent affinity, markedly enhanced binding to membrane-bound CD40L, and potent inhibition of CD40L-induced NF-κB activation in a reporter cell assay. Collectively, these results establish collagen XV and XVIII NC1 domains as effective trimerization modules for affibody molecules and highlight a strategy for engineering Fc-independent, multivalent antagonists targeting CD40L.

**Graphical Abstract:**

**Figure:**
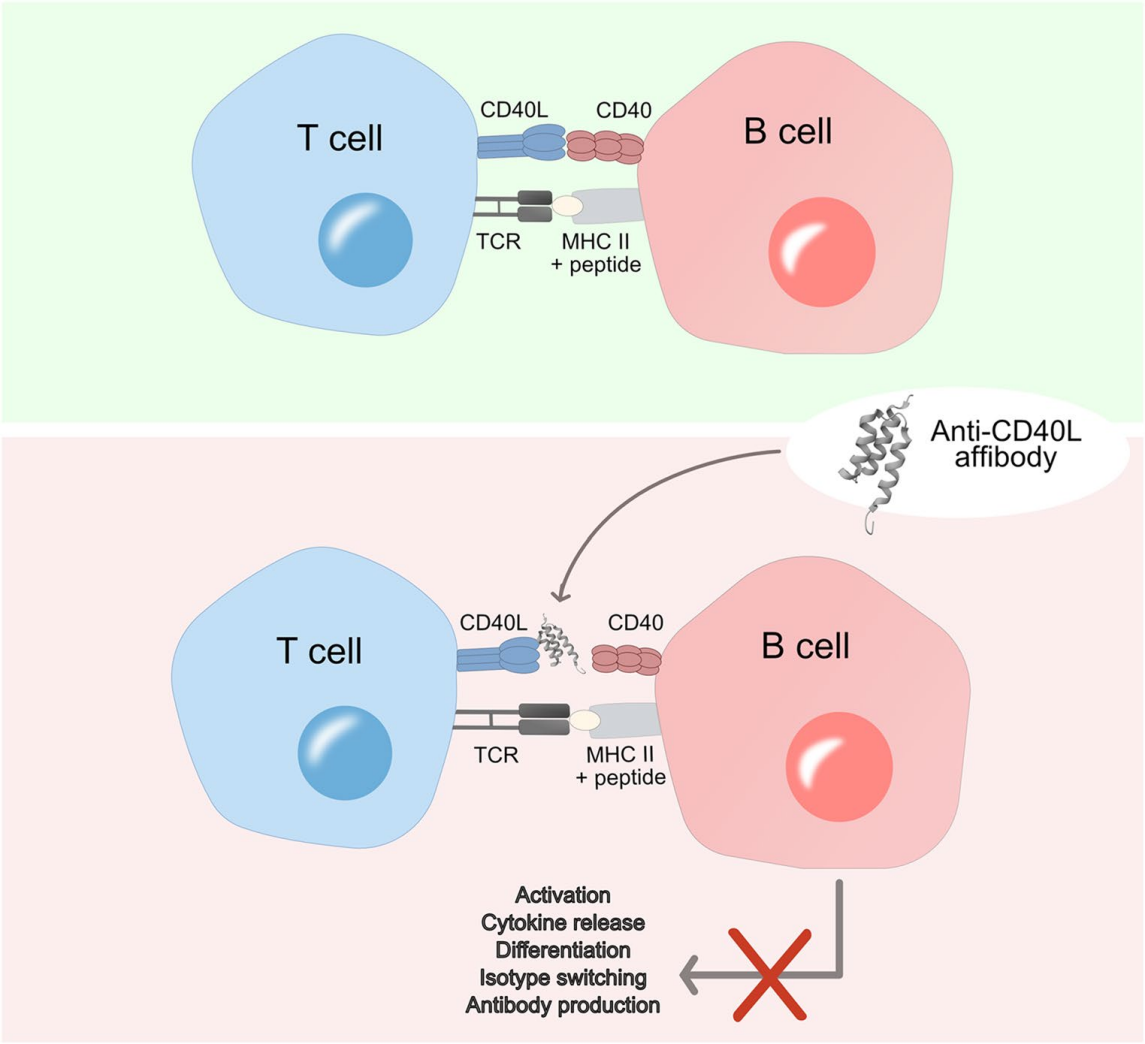

**Supplementary Information:**

The online version contains supplementary material available at 10.1007/s00018-026-06301-2.

## Introduction

The CD40/CD40 ligand (CD40L) costimulatory pathway plays a central role in the regulation of adaptive immune responses. Through engagement of CD40 on antigen-presenting cells (APCs), CD40L provides essential signals that govern immune activation, differentiation, and memory formation. Dysregulation of this pathway has been implicated in a wide range of inflammatory and autoimmune diseases, as well as in transplant rejection, making CD40L an attractive therapeutic target for immune modulation [1, 2].

CD40 is a type I transmembrane protein belonging to the tumor necrosis factor receptor (TNFR) family [3]. Its cognate ligand, CD40L, is a member of the tumor necrosis factor (TNF) superfamily and comprises an N-terminal intracellular domain, a short transmembrane segment, a 65-residue extracellular stalk, and a C-terminal extracellular domain of approximately 150 residues. The extracellular domain adopts a sandwich-like fold consisting of two β-sheets and an α-helix loop containing a conserved TNF homology domain, which mediates noncovalent assembly into stable homotrimers [4, 5]. This trimeric organization enables multivalent CD40 ligation, which promotes receptor clustering and downstream signaling via recruitment of TNFR-associated factors (TRAFs) and activation of NF-κB, MAPK, PI3K, and PLCγ pathways [3]. Constitutive expression of CD40 was first identified on B cells and was later also demonstrated on other professional APCs, including dendritic cells (DCs) and macrophages. In addition, CD40 expression has been reported on non-hematopoietic cells such as fibroblasts, endothelial cells, and epithelial cells [3]. CD40L expression is tightly regulated and is primarily detected on activated CD4⁺ T cells, but is also present on activated platelets and B cells ^[^ [6–8]. Inducible expression has further been reported in mast cells, basophils, eosinophils, monocytes, natural killer (NK) cells, as well as in vascular endothelial cells and smooth muscle cells, under inflammatory conditions [3, 9, 10]. Both CD40 and CD40L exist in membrane-bound and soluble forms following proteolytic processing [11]. Similar to membrane-bound CD40L, soluble CD40L forms trimers and retains the ability to activate CD40 [12]. Extensive evidence demonstrates that the CD40/CD40L interaction is critical for the development of adaptive immune responses [3, 13]. In humoral immunity, CD40 ligation on B cells by CD40L expressed on CD4⁺ T cells is required for immunoglobulin class switching, affinity maturation, and the establishment of humoral immune memory [3]. Disruptive mutations in the CD40L gene result in hyper-IgM syndrome, a primary immunodeficiency characterized by impaired immunoglobulin class switching [14]. In vivo studies have further shown that CD40/CD40L interactions are required for germinal center formation, B cell proliferation and differentiation, and the generation of memory B cells and long-lived plasma cells [3, 15]. In addition, CD40 signaling plays a key role in cell-mediated immunity by enabling activation and maturation of CD40-expressing, alloantigen-presenting DCs [16]. Disruption of this interaction can result in abortive activation and anergy of alloreactive T cells [3].

Despite its therapeutic potential, early clinical efforts to target CD40L were hindered by severe adverse events. Fatal thromboembolic complications were observed in clinical trials using an IgG1 anti-CD40L antibody, which were attributed to Fc-mediated crosslinking of CD40L and Fcγ receptors on platelets [1, 17]. These outcomes prompted the development of next-generation CD40L antagonists incorporating Fc-silencing mutations or lacking Fc domains altogether. Several such agents have since advanced to late-stage clinical trials with favorable safety profiles, reviving interest in CD40L as a therapeutic target for autoimmune diseases [1].

Affibody molecules are non-immunoglobulin derived proteins with several properties that distinguish them from antibodies, while sharing their capacity to reach high target affinity and specificity. The affibody scaffold (denoted Z) is engineered from the IgG-binding domain B from *Staphylococcus aureus* protein A and is composed of 58 amino acids forming three alpha helices [18]. Their small size (~ 6.5 kDa), lack of inherent cysteines and ease of production in prokaryotic hosts or by chemical synthesis make affibody molecules highly modular and favorable tools in biomedical engineering [19]. Importantly, affibody molecules inherently lack Fc domains, making them attractive candidates for targeting CD40L.

The objective of this study was to develop CD40L-specific affibody molecules capable of inhibiting the CD40/CD40L interaction. To this end, CD40L-binding affibody variants were isolated by directed evolution using an in-house developed *Escherichia coli* surface display platform combined with flow-cytometry sorting [20]. Guided by the homotrimeric architecture of CD40L, we further explored whether functional performance could be enhanced by matching binder valency to target structure through protein-based trimerization. Selected affibody candidates were genetically fused to the NC1 domains of human collagen XV or XVIII. These domains were selected based on previous studies demonstrating their ability to form stable homotrimers as independent domains and their successful use as compact trimerization modules in recombinant fusion proteins [21–24]. Compared with other collagen assembly systems that rely on supramolecular fibril or network formation, collagen XV and XVIII NC1 domains efficiently form stable trimers as isolated domains, making them well suited for protein engineering applications [25]. In addition, these human-derived domains are relatively small, predicted to exhibit low immunogenicity, and lack disulphide bonds, which facilitates production in prokaryotic hosts [21]. The resulting fusion proteins were evaluated with respect to oligomeric state, thermal stability, binding kinetics, cellular target engagement, and inhibition of CD40 signaling. By combining directed evolution with rational multimerization, this study investigates a strategy to convert weakly inhibitory monovalent binders into potent, Fc-independent antagonists of CD40L.

## Materials & methods

### *E. coli* display

Luria-Bertani (LB) medium supplemented with 0.1 mg/mL carbenicillin (LB-carb100) was inoculated with BL21* *Escherichia coli* (*E. coli*) carrying the arabinose-inducible pPALU1 [20] vector containing affibody-encoding genes genetically fused to an albumin binding domain (ABD_035_) [26]. Cultures were incubated for approximately 16 h at 37°C and 150 rotations per minute (rpm). The next day, they were diluted at 1:100 and cultivated until the optical density at 600 nm (OD_600_) was between 0.5 and 0.8, whereupon they were induced by adding L-arabinose up to 0.6%. Induced cultures were incubated at 25°C and 150 rpm for 16 h. Affibody-displaying *E. coli* cells were harvested and dissolved in phosphate-buffered saline with 0.1% w/v pluronic acid (PBS-P) and utilized for downstream experiments.

### Magnetic-activated cell sorting

A naive affibody library in BL21* *E. coli*, of library size 1.5 × 10^11^, was used for the selections described herein. For the first round of magnetic-activated cell sorting (MACS), a volume of glycerol stock corresponding to 6x the naive library size was inoculated in 4 L LB-carb100. For practical reasons, the volume was divided into eight 5 L E-flasks. Before induction, a volume corresponding to 1/8 of the library size was taken from each flask to pool the library in 1 L media, which was then divided into two 5 L E-flasks for subsequent induction. The induced cultures were measured for OD_600_ after 16 h and an amount corresponding to ½ of the naive library size was taken from each flask to make up the cellular input to the selection.

A volume of Dynabeads™ MyOne™ Streptavidin C1 beads (cat.no. 65001, ThermoFisher) corresponding to 1/50 of the cell amount was used for positive selections and approximately half of that bead amount was used for negative selections. For round one of MACS, the beads were washed twice with 20 mL PBS-P using magnetic separation. The beads used for positive selection were incubated with 20 µg biotinylated trimeric human CD40L (hCD40L, cat.no. CDL-H82Db, Acro Biosystems) in 20 mL PBS-P for 1 h with rotation (150 rpm) at room temperature (RT). Following this, the beads were washed with 20 mL PBS-P twice using magnetic separation.

The cells were washed three times with 50 mL cold PBS-P and subsequent pelleting by centrifugation (4000xg, 10 min, 4 °C). They were then resuspended in 20 mL PBS-P with washed, naked beads and incubated for 30 min with rotation (150 rpm) at RT. Following this, the beads were separated on a magnetic rack and the supernatant was added to the target-bound beads. The suspension was incubated for 2 h with 150 rpm rotation at RT, whereafter the beads were separated on a magnetic rack for 10 min. The beads were washed three times with 10 mL cold PBS-P and the remaining cells and beads were then pelleted by centrifugation (4000xg, 10 min, 4 °C) before resuspension in 50 mL LB-carb100. A volume of 10 µL was taken for serial dilutions to titrate the output and estimate the library size. The remaining volume was incubated at 37 °C, 150 rpm, for 16 h. The next day, OD_600_ was measured, and glycerol stocks of the output library were prepared for long-term storage at −80 °C.

The subsequent two rounds of MACS followed the same protocol with the following modifications: The library size of the previous round was covered by 20x and 100x for round two and three, respectively. The volume required for library cultivation was reduced to 10 mL and the wash volumes for beads and cells were reduced to 500 µL and 10 mL PBS-P, respectively. The concentration of biotinylated CD40L varied from 20 nM in round one (20 mL) to 50 nM in round two (500 µL) and 30 nM in round three (250 µL), while keeping the bead-to-cell ratio constant at 1:50.

After each cycle, the output library was titrated on agar plates for estimation of library size and glycerol stocks were prepared for long term storage.

### Fluorescent-activated cell sorting

Library preparations were performed according to the MACS protocol, with each selection cycle using 100–1000× coverage of the preceding output library size. Induced cells were washed twice by centrifugation (6000 rpm, 6 min, 4 °C) in PBS-P and then resuspended in 100 µL biotinylated hCD40L with concentrations ranging from 100 nM to 5 nM. The target incubation was performed at RT and at 150 rpm. Thereafter, cells were washed twice and resuspended in PBS-P for an off-rate incubation, consisting of three 20-minute incubations in PBS-P with washes between each incubation, performed at RT. The off-rate incubations were performed for the first and second round of fluorescent-activated cell sorting (FACS, hereafter referred to as FACS 1 and FACS 2).

Subsequently, the cells were labeled on ice with 200 µL HSA-AlexaFluor 647 (333 nM) (prepared in-house) and Streptavidin Phycoerythrin (SAPE) or Neutravidin Phycoerythrin (NAPE) (cat.no. S866 and cat.no. A2660, Thermo Scientific, respectively), both at 2 µg/mL, for 30 min. After two washes and resuspension in PBS-P, the cells were sorted on a Cytoflex SRT (Beckman Coulter, Indianapolis, IN, USA) with laser/filter combinations of 561/543–627 nm and 638/650–670 nm, gating manually for the top binding fraction of cells. All output populations were phenotyped in LB for 1 h at 37 °C, before cultivating them in LB-carb100 for approximately 16 h. Glycerol stocks were prepared after each cycle for subsequent selections and analysis. Plated clones from FACS 3 were sent for sanger sequencing at Microsynth AG (Balgach, Switzerland) and unique clones were cultivated and analyzed by flow cytometry in *E. coli* display.

### Next generation sequencing of sublibraries

Glycerol stocks of the output libraries from MACS 3, FACS 1, FACS 2 and FACS 3 were cultivated overnight in LB-carb100, and the cultures were subjected to miniprep with QIAprep^®^ Spin Miniprep Kit (cat.no. 27106, QIAgen). Following PCR with barcoded primers to amplify the affibody-encoding DNA, gel extraction performed using the QIAquick^®^ Gel Extraction Kit (cat.no. 28706, QIAgen). An agarose gel was run to verify the size and purity of the products and the DNA concentrations were measured on a NanoDrop (model ND-1000, Thermo Scientific), whereafter they were pooled in equal amounts. The pooled DNA was sequenced using MiSeq (National Genomics Infrastructure, Stockholm, Sweden) and analyzed with PipeBio (Horsens, Denmark). Sequences in each output library were annotated, filtered and subsequently clustered on 100% identity to reveal unique clones and their respective frequencies in the libraries. Clonal enrichment over the different selection cycles was analyzed for the thirty most enriched variants.

### High-throughput single clone screen using *E. coli* display

Unique clones from FACS 3 (determined by Sanger sequencing) were inoculated from glycerol stocks and cultivated overnight in 200 µL LB-carb100. The cultures were induced using the same protocol as that used during the selections. Five µL of each induced culture was added to 200 µL PBS-P in a 96-well conical plate. The cells were washed twice by centrifugation (2000xg, 2 min, 4 °C) and resuspension in 200 µL PBS-P. Thereafter, they were resuspended in 0.5–100 nM hCD40L or murine CD40L (mCD40L) (cat.no. CDL-M82H5 Acro Biosystems) and incubated with 150 rpm shaking at RT for 1 h. After the target incubation, the cells were washed twice and subsequently labeled on ice with 100 µL HSA-AlexaFluor 647 (333 nM) and SAPE 2 µg/mL. After the secondary labeling, the cells were washed twice and resuspended in 200 µL PBS-P before analyzing > 50 000 events in a CytoFlex S (Beckman Coulter, Indianapolis, IN, USA) with laser/filter combinations of 561/543–627 nm and 638/650–670 nm.

### Sequence library filtering and selection of candidates for soluble production

Clone selection for downstream soluble production was based on two parameters evaluated simultaneously during display screening: (i) detectable surface expression and (ii) binding to recombinant hCD40L. These properties were assessed using fluorescence gating on the expression channel (638/650–670 nm) and binding channel (561/543–627 nm), respectively. Clones exhibiting fluorescence signals above the gate thresholds in both channels were considered positive. Candidate selection was subsequently guided by comparative assessment of binding signal relative to display level. No filtering based on enrichment frequency or enrichment trajectory across selection rounds was applied.

### CD40L specificity assay

To assess target specificity, the lead affibody candidates D10 and G6 were tested for binding to hCD40L along with three unrelated proteins: human PD-1, human CD161 and human 4-1BB (cat.no. PD1-H82E4, CD1-H82E3 and 41B-H82E6, respectively, Acro Biosystems). The assay was performed in technical triplicates according to the same protocol described above for the high-throughput single-clone screen using the *E. coli* display format and the same concentration of CD40L as the unrelated proteins (100 nM).

### Production and purification of soluble affibody molecules

Affibody candidates selected for production and characterization in soluble format were subcloned into the pET45b(+) production vector (Novagen), containing an isopropyl β-D-1-thiogalactopyranoside (IPTG)-inducible T7 promoter, an AmpR gene and a C-terminal hexahistidine tag, using In-Fusion^®^ Snap Assembly Master Mix (cat.no. 638949, Takara Bio) according to the manufacturer’s instructions. Transformants were Sanger sequenced at Eurofins Genomics (Ebersberg, Germany). Affibody clones (Z-H_6_) were cultivated overnight in TSB with yeast extract (TSB + Y) supplemented with 0.1 mg/mL carbenicillin and subsequently diluted 1:100 TSB + Y supplemented with 0.1 mg/mL carbenicillin. The cultures were grown until reaching 0.6 ≤ OD_600_ ≤ 1.0, whereupon they were induced by adding IPTG to a final concentration of 1 mM. The induced cultures were incubated at 25 °C and 150 rpm overnight before harvesting and lysis by sonication. Lysates were purified through immobilized metal affinity chromatography (IMAC) at 4 °C using HisPur Cobalt Resin (cat.no. 89966, ThermoFisher) dispensed in 15 mL Falcon tubes for batch purification and native conditions were used according to the manufacturer’s recommendations. The eluates were buffer exchanged to 1xPBS pH 7.4 using PD-10 columns (cat.no. 17085101, Cytiva) according to the manufacturer’s recommendations. Purified proteins were analyzed for purity by sodium dodecylsulphate-polyacrylamide gel electrophoresis (SDS-PAGE) (cat.no. NP0321BOX, Invitrogen) and concentration by bicinchoninic acid assay (cat.no. 23227, ThermoFisher).

### Surface plasmon resonance to evaluate binding of soluble candidates

Affibody candidates were screened by surface plasmon resonance (SPR) to identify binders following selections on a Biacore 8 K (Cytiva, Uppsala, Sweden). A Series S sensor chip CM5 (cat.no. 29149603, Cytiva) was immobilized with recombinant trimeric human CD40L (cat.no. CDL-H82Db, Acro Biosystems) and murine CD40L (cat.no. CDL-M82H5, Acro Biosystems) dissolved in 10 mM pH 5.5 NaAc using Cytiva’s amine coupling kit (cat.no. BR100050) and aiming for 1000 response units (RU).

The candidates were diluted in 1xPBS pH 7.4 supplemented with 0.5% Tween (PBS-T) to concentrations ranging from 1000 nM to 0 nM and injected over the target immobilized chip surface for 200 s, followed by dissociation for 400 s. Running buffer was PBS-T and the analysis temperature was constant at 25 °C. To regenerate the surface between cycles, 10 mM HCl was injected for 30 s.

### Binding of affibody candidates to human primary T cells

1 × 10^6^ human peripheral blood mononuclear cells (PBMCs)/well were stimulated with 0.05 mg/ml phorbol myristate acetate (PMA) and 1 mg/ml ionomycin for 4 h at 37 °C, in a total volume of 200 ml/well complete RPMI with 10% fetal bovine serum. Subsequently, cells were washed and incubated with 1:1000 Human Fc block (cat.no. 564219, BD) and 1:500 LIVE/DEAD™ Fixable Yellow Stain (cat.no. L34959, ThermoFisher) for 15 min at 4 °C. After another wash, cells were incubated for 20 min at 4 °C with: 1:50 anti-CD40L PE (cat.no. 555700, BD), 1:100 anti-CCR7 PE-Cy7 (cat.no. 353226, BioLegend), 1:100 anti-CD45RA BV421 (cat.no. 562885, BD), 1:100 anti-CD3 APC-H7 (cat.no. 560176, BD), 1:200 anti-CD4 Qdot800 (cat.no. Q22153, Invitrogen). Cells were then washed and incubated with 2 mM affibody candidates for 30 min at room temperature, followed by a 20-minute staining with 1:2000 anti-6x-His Tag antibody Alexa Fluor 647 (cat.no. MA1-21315-A647, Invitrogen) at 4 °C. Data were acquired on a BD Fortessa instrument with gating strategy shown in Figure S1 (Supplementary) and analyzed with FlowJo software.

### Evaluation of secondary structure content and thermal stability by circular dichroism spectroscopy

Candidate affibodies were diluted to 0.1–0.3 mg/mL in PBS (pH 7.4) and analyzed using a Chirascan Circular Dichroism Spectrometer (Applied Biophysics Ltd, Leatherhead, UK) with a 1 mm path length cuvette. To assess secondary structure content, ellipticity spectra were recorded from 195 to 260 nm, averaging over five scans. Thermal denaturation was then performed by increasing the temperature from 20 °C to 95 °C at a rate of 5 °C/min, while monitoring ellipticity at 221 nm. The thermal melting point (T_m_) was calculated as the inflection point of a four-parameter sigmoidal curve fitted to the temperature-dependent data using GraphPad Prism 10 (Dotmatics, Boston, MA, USA). After cooling the samples to below 25 °C, CD scans were repeated to evaluate the refoldability of the affibodies.

### Trimerization of affibody molecules using collagen domains

Affibody molecules were reformatted to homotrimeric constructs by genetically fusing them to the N-terminal trimerization region of collagen XV or collagen XVIII NC1 domains [25, 27] (sequences shown in Table S1, Supplementary), either in the N- or C-terminus, incorporating a N-terminal hexahistidine (H_6_) tag and a flexible (G_4_S)_4_ linker between the affibody and the collagen domain. Four prototype gene fragments, H_6_-collagenXV-(G_4_S)_4_-Z_wt_, H_6_-collagenXVIII-(G_4_S)_4_-Z_wt_, H_6_-Z_wt_-(G_4_S)_4_-collagenXV and H_6_-Z_wt_-(G_4_S)_4_-collagenXVIII comprising a non-CD40L specific affibody (Z_wt_) were ordered from Twist Bioscience (South San Francisco, CA, USA) and were cloned into an *E. coli* expression vector. The Z_wt_ domain was subsequently exchanged to the different affibody candidates using In-Fusion^®^ Snap Assembly Master Mix (cat.no. 638949, Takara Bio) according to the manufacturer’s instructions. Plated clones were picked and sent for Sanger sequencing. Protein production and purification of sequence-verified constructs was done as previously described for the monomeric candidates. Purified proteins were analyzed by SDS-PAGE (NuPAGE, cat.no. NP0329BOX, Invitrogen) and concentrations were measured in a Qubit 4 Fluorometer (Invitrogen, Waltham, MA, USA), using Qubit™ Protein BR Assay Kit (cat.no. A50669, Invitrogen) according to the manufacturer’s recommendations. To assess oligomeric state, affibody monomers and trimers were analyzed by size exclusion chromatography (SEC), using a Superdex 75 Increase 5/150 GL column (cat.no. 29148722, Cytiva) and a Gel Filtration Calibration Kit LMW (cat.no. 28403841, Cytiva) according to the manufacturer’s recommendations. The trimeric constructs were analyzed for secondary structure content and thermal stability by CD spectroscopy as previously described.

### Kinetic screen of affibody monomers and trimers

Selected clones were assessed kinetically as monomers and collagen NC1-mediated homotrimers by SPR on a Biacore T200 (Cytiva, Uppsala, Sweden). A Series S sensor chip CM5 (cat.no. 29149603, Cytiva) was immobilized with 800 RU of recombinant human CD40L (cat.no. CDL-H82Db, Acro Biosystems).

The running buffer was 10 mM HEPES buffered saline, 150 mM NaCl, 3 mM EDTA, 0.005% v/v Tween-20 at pH 7.4 (HBS-EP). The proteins were diluted in HBS-EP at concentrations ranging from 1000 − 31.25 nM for monomers and 125 − 3.9 nM for trimers, and these were injected for 400 s, followed by 1000 s dissociation. To regenerate the chip surface between cycles, a solution of 10 mM NaOH + 200 mM NaCl + 0.2% SDS was injected for 30 s. A 1:1 Langmuir curve fitting model was used to estimate kinetic parameters of both monomers and trimers in the Biacore T200 software.

### Surface plasmon resonance assay to evaluate CD40 blocking

A Series S sensor chip CM5 (cat.no. 29149603, Cytiva) was immobilized with 580 RU of recombinant human CD40 (cat.no. TN5-H82F9, Acro Biosystems). Candidate proteins were serially diluted in running buffer (HBS-EP at pH 7.4) and recombinant trimeric human CD40L (cat.no. CDL-H52Db, Acro Biosystems) was added to each sample to a final concentration of 50 nM. The samples were incubated at RT for 45 min, before injecting the samples sequentially over the chip surface for 150 s, followed by dissociation for 600 s. The surface was regenerated between cycles using a 30 s pulse of 10 mM Glycine-HCl pH 2.5. The R_eq_ values were obtained by averaging the binding signal over 145–150 s. Sigmoidal CD40-blocking curves were achieved by plotting the R_eq_ values against the candidate concentrations and normalizing separately for each candidate.

### Flow cytometry analysis of monomeric and trimeric affibody interactions with mammalian cells

Jurkat wildtype cells (ACC282, DSMZ, Germany) and Jurkat D1.1 cells (ATCC-CRL-3600, LGC Nordic, UK) were grown in 90% RPMI 1640 Medium (cat.no. 21875, Gibco) and 10% heat-inactivated fetal bovine serum (cat.no. 17914671, Gibco) at 37 °C, 5% CO_2_ and subcultured according to manufacturer’s recommendations.

Approximately 200 000 cells per sample were harvested and washed twice by centrifugation at 300xg, 4 min, with 5 mL PBS (pH 7.4) supplemented with 0.1 w/v% bovine serum albumin (cat.no. A4503, Sigma-Aldrich, USA) (PBS-B). After the wash, the cells were diluted to a volume corresponding to ≤ 2.0 × 10^6^ cells/mL and 100 µL of the cell suspension was added to each sample in a conical 96-well plate. The cells were pelleted by centrifugation (600xg, 2 min) and resuspended in 100 µL affibody candidate, serially diluted in PBS-B with concentrations ranging from 4 to 500 nM. Labeling proceeded for 1 h at RT and 150 rpm. The cells were then pelleted by centrifugation (600xg, 2 min) and washed once with 200 µL PBS-B. After re-pelleting by centrifugation, they were resuspended in 100 µL anti-Z murine IgG (proprietary of Affibody AB, Stockholm, Sweden), diluted to 6 µg/mL in PBS-B. The secondary labeling proceeded at 4 °C and 150 rpm for 45 min, whereupon the cells were again pelleted and washed once. Fluorescent labeling followed by resuspending pelleted cells in 100 µL anti-mIgG monoclonal antibody Alexa647 conjugate (cat.no. A21236, Invitrogen) diluted 1:500 in PBS-B or anti-hCD40L monoclonal antibody PE conjugate (cat.no. 12–1548-42, Invitrogen) diluted 1:100. The fluorescent labeling reaction proceeded for 45 min on ice. Following the reaction, cells were washed once before resuspension in 200 µL PBS-B for analysis. A minimum of 10 000 events was recorded by a Cytoflex S (Beckman Coulter, Indianapolis, IN, USA) with laser/filter combinations 488/525–40 nm and 638/660–10 nm.

### NF-κB reporter cell assay for CD40 inhibition

HEK-Blue™ CD40L cells (cat.no. hkb-cd40, Invivogen) were grown according to manufacturer’s recommendations. For the assay, approximately 50 000 cells per sample were collected from the culture and these were centrifuged at 300xg, 5 min. The culturing media was discarded and the cells were resuspended in test media to a final concentration of approximately 280 000 cells/mL.

Candidate and negative control constructs were serially diluted in test media supplemented with 5 nM recombinant trimeric human CD40L (cat.no. CDL-H82Db, Acro Biosystems). The candidate concentrations were 2500, 500, 100, 20, 4, 0.8, 0.16, 0.03, and 0 nM, which was used as positive control for all samples. As negative controls for cell reactivity, each candidate was also diluted in test media without CD40L. Additional negative controls for cell reactivity were test media supplemented with 10 nM recombinant IL-6 and test media only. The dilutions were incubated for 45 min at RT with rotation. After the pre-incubation, 20 µL of the respective samples was dispensed in triplicate wells of a 96-well plate. 180 µL cell suspension was then added to the wells for a final volume of 200 µL per sample. The plates were incubated at 37 °C and 5% CO_2_ for 20 h.

QUANTI-Blue ^TM^ (cat.no. rep-qbs, Invivogen) solution was prepared according to the manufacturer’s instructions and 180 uL of the solution was dispensed in the wells of a 96-well plate. 20 µL of each sample supernatant was added to the solution and carefully pipette-mixed. The plate was placed at 37 °C and 5% CO_2_ for 10–20 min, before analyzing the absorbance at 620 nm. Obtained values were processed in GraphPad Prism 10 (Dotmatics, Boston, MA, USA), where they were averaged and normalized to the averages of the positive control wells (0.5 nM recombinant CD40L) and the negative control (test media only). A dose-response curve using a four-parameter sigmoidal curve fit was applied to the normalized data in order to obtain IC_50_.

## Results

### Selections for CD40L-targeting affibody molecules

**Fig. 1:**
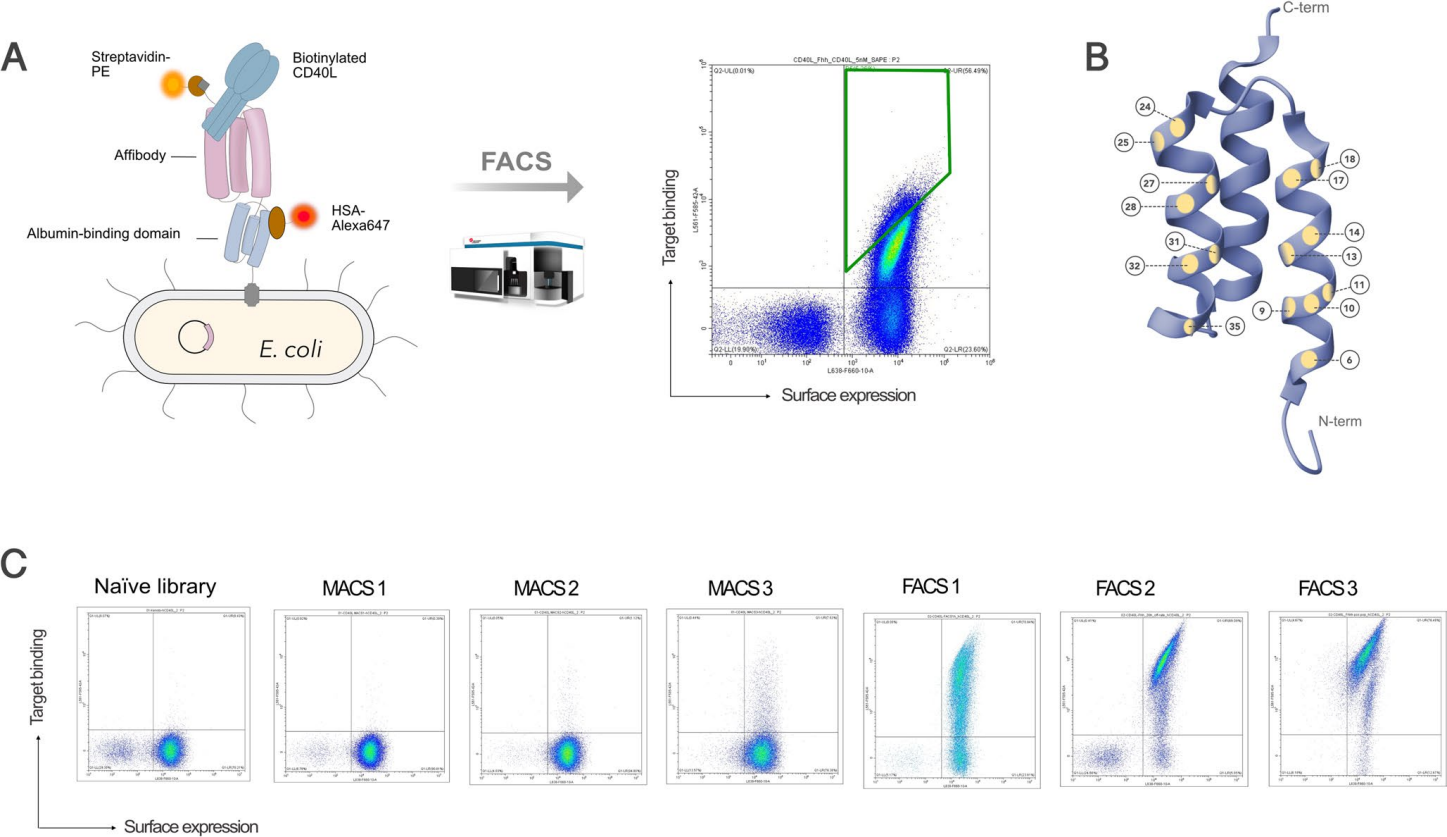
**(A)** Schematic representation of the *E. coli* display system used for selections. The surface-expressed fusion protein consists of an albumin-binding domain for expression normalization and an affibody library member. Cells displaying affibody variants capable of binding biotinylated CD40L were isolated by fluorescent-activated cell sorting (FACS). The gate used for sorting is indicated in green. The x-axis shows fluorescence intensity corresponding to surface display (561/543–627 nm), and the y-axis shows fluorescence intensity corresponding to hCD40L binding (638/650–670 nm). **(B)** The protein structure of an affibody molecule with diversified positions indicated in yellow. **(C)** Flow cytometry dot plots from analysis of the naive library and the output libraries from successive selection rounds, comprising three rounds of MACS, followed by three rounds of FACS. Each plot represents one library, with axes corresponding to surface display (x-axis) and hCD40L binding (y-axis)

To generate affibody molecules targeting human CD40L, directed evolution was performed using an in-house developed *Escherichia coli* surface display system [20] (Fig. 1A). A naive affibody library comprising approximately 1.5 × 10¹¹ variants, diversified at 15 positions within the binding surface (Fig. 1B), served as the starting point for selections against recombinant, biotinylated human trimeric CD40L. The selection strategy consisted of three rounds of magnetic-activated cell sorting (MACS) followed by three rounds of fluorescent-activated cell sorting (FACS). During the MACS rounds, the library was challenged with increasing stringency while maintaining a constant bead-to-cell ratio, resulting in progressive enrichment of CD40L-binding populations. Analysis of output libraries after each MACS round confirmed successful enrichment of CD40L-binding cells. Following MACS enrichment, the output library was subjected to three successive rounds of FACS. In these rounds, cells were incubated with soluble biotinylated CD40L and analyzed by dual-color flow cytometry, allowing simultaneous assessment of surface expression level via the albumin-binding domain and fluorescently labelled albumin, as well as target binding via streptavidin-PE labeling. Sorting gates were set to capture the top binding fraction and flow cytometry analysis revealed a progressive shift of the population toward increased CD40L-binding over successive selection rounds (Fig. 1C). Distinct CD40L-positive populations emerged after MACS enrichment and became increasingly pronounced during FACS rounds, culminating in a well-defined high-binding population after the third FACS round.

To assess clonal diversity and enrichment following selection, individual clones from the FACS output were analyzed by Sanger sequencing, revealing sequence diversity among the isolated clones. In parallel, next-generation sequencing was performed on output libraries from MACS round 3 and all three FACS rounds to enable quantitative tracking of clonal enrichment. Illumina MiSeq sequencing confirmed progressive enrichment of variants over successive rounds, while still retaining a diverse population (Figure S2, Supplementary). Among the enriched clones, variant D10 emerged as the most prominent sequence, accounting for approximately 30% of the Sanger-sequenced clones from the final output. In the MiSeq dataset, D10 represented approximately 7% of total reads in the FACS round 3 library (Figure S3, Supplementary). Several additional variants demonstrated clear enrichment trends across MACS and FACS rounds, suggesting the presence of multiple CD40L-binding solutions within the selected population.

### Hits screening and characterization

**Fig. 2:**
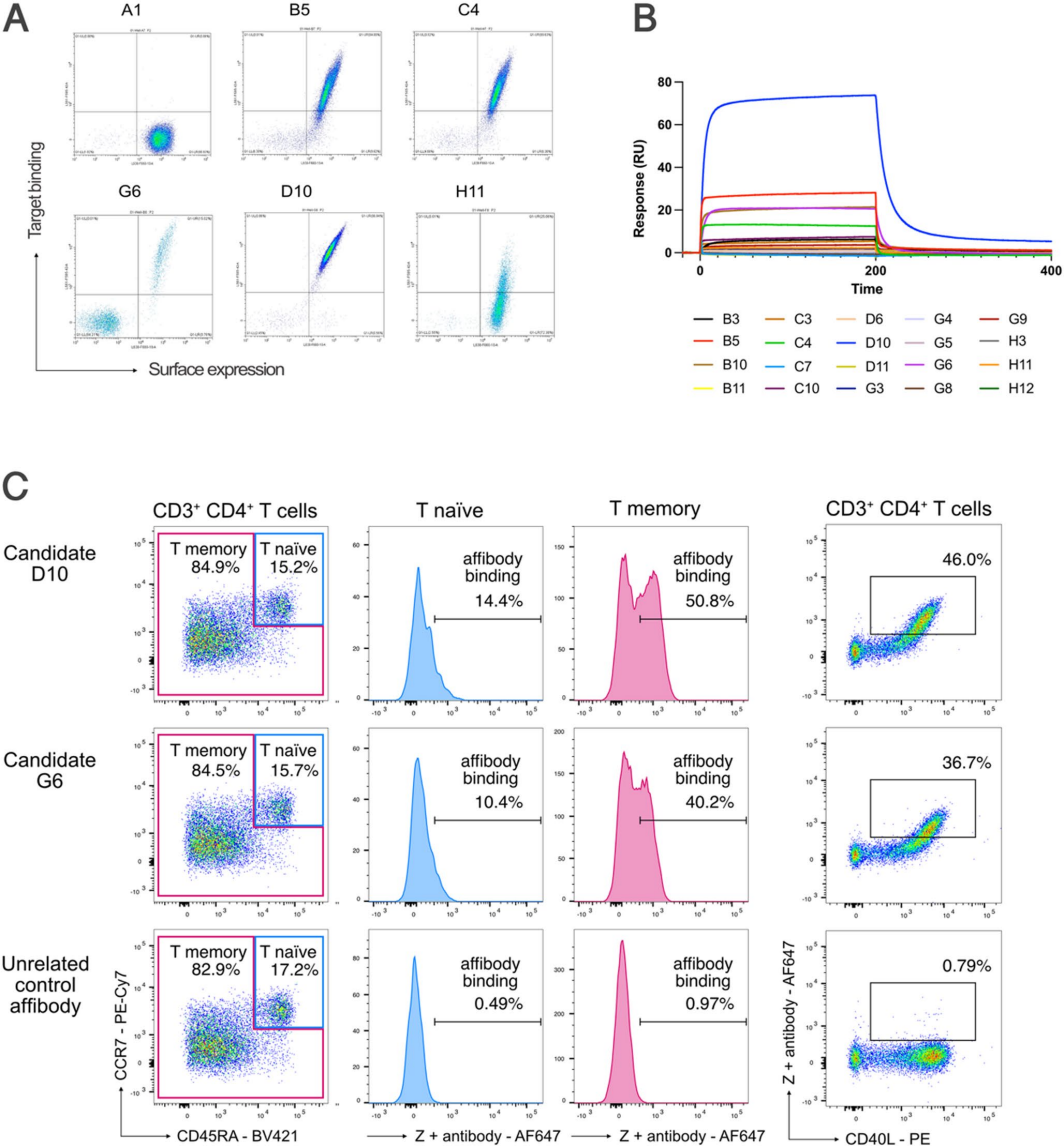
**(A)** Representative flow cytometry dot plots from high-throughput screening of unique affibody clones in the *E. coli* display format. Each dot plot corresponds to a unique clone, with axes representing surface display (x-axis) and hCD40L binding (y-axis) as described above. **(B)** SPR sensorgrams from soluble protein candidates, indicating their different abilities to bind to recombinant hCD40L immobilized on the chip surface. **(C)** Flow cytometry analysis showing binding of candidate or control affibodies and an anti-CD40L antibody to CD3^+^CD4^+^ T cells from human peripheral blood following in vitro stimulation with phorbol myristate acetate (PMA) and ionomycin

Single clones identified from the final sorting output were first screened by flow cytometry in the *E. coli* display format to confirm CD40L binding (Fig. 2A). Screening was performed using a high-throughput single-clone assay, enabling comparative assessment of surface-displayed affibody variants. Several clones displayed signals above background, confirming successful enrichment of CD40L-binding variants during the selection. By comparative assessment of binding signal relative to display level, twenty unique clones were selected for production and characterization in soluble format. The selected affibody variants were expressed in *E. coli* and purified by immobilized metal affinity chromatography (IMAC). Binding of the soluble affibody candidates to recombinant trimeric human CD40L was evaluated using surface plasmon resonance (SPR) (Fig. 2B). Thirteen candidates displayed detectable binding to immobilized CD40L under the screening conditions. The interaction profiles were characterized by relatively rapid association and dissociation phases, indicative of fast binding kinetics. SPR data thus confirmed that all selected candidates retained CD40L binding activity upon conversion from the surface-displayed to the soluble format.

### T cell binding

The affibody candidates were further evaluated for their ability to recognize CD40L in a cellular context using human primary T cells. PBMCs were stimulated in vitro to induce CD40L expression and subsequently incubated with soluble affibody candidates. Among the screened variants, two candidates, D10 and G6, bound to stimulated T cells (Fig. 2C), while no binding to unstimulated T cells was observed for these candidates (Figure S4, Supplementary). Flow cytometry analysis revealed that both D10 and G6 preferentially stained memory T cells, whereas binding to naive T cells (CD45RA⁺ CCR7⁺) was markedly lower. This distribution is consistent with previous reports showing that naive T cells have a reduced capacity to rapidly mobilize preformed intracellular CD40L to the plasma membrane upon stimulation, compared with effector and memory T cell subsets [28]. To confirm target specificity, stimulated PBMCs were co-stained with the affibody candidates and a CD40L-specific monoclonal antibody. Binding of both D10 and G6 closely correlated with antibody staining, with affibody-positive cells corresponding to populations exhibiting high CD40L expression levels (Fig. 2C).

### Secondary structure content, thermostability and binding kinetics

**Fig. 3:**
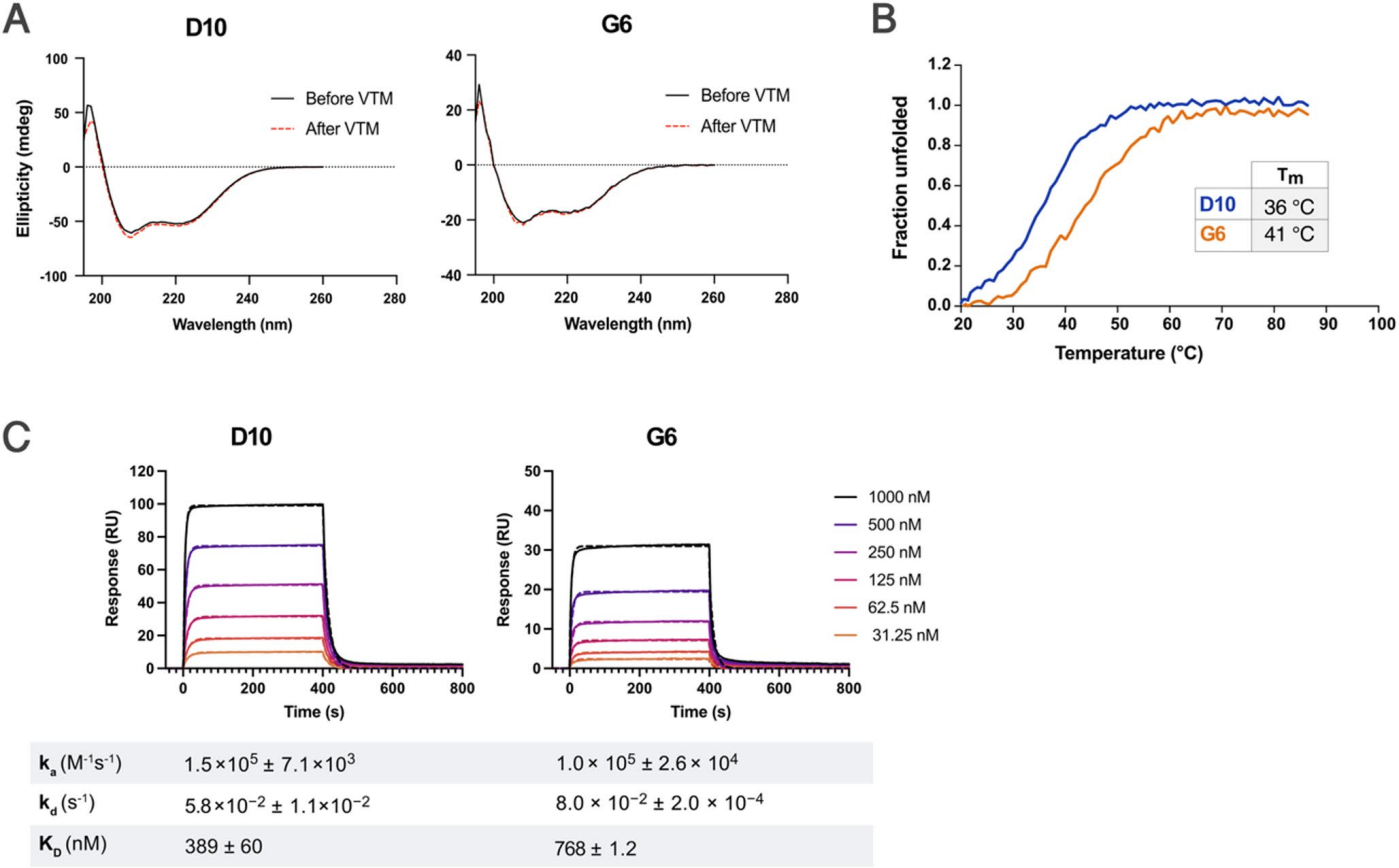
Biophysical characterization and binding kinetics of monomeric CD40L-binding affibody candidates.** (A)** Circular dichroism (CD) spectra of the affibody candidates D10 and G6 recorded at room temperature before (solid lines) and after (dashed red lines) variable temperature measurements (VTM). **(B)** Melting curves from the VTMs of D10 (blue) and G6 (orange) with indicated melting temperatures. **(C)** Sensorgrams from kinetic assessment of D10 and G6 with recombinant hCD40L immobilized on the chip surface. The respective kinetic constants are shown in the bottom table with values representing four independent measurements

The biophysical properties of the lead affibody candidates D10 and G6 were further characterized with respect to secondary structure content, thermal stability, and refoldability using circular dichroism (CD) spectroscopy (Fig. 3A). Far-UV CD spectra recorded at room temperature revealed profiles dominated by α-helical secondary structure for both candidates, consistent with the expected folding of the affibody scaffold. The spectra before and after variable temperature measurement (VTM) overlapped well, indicating the capacity of both D10 and G6 to refold completely after heat-induced denaturation. The melting temperatures of both candidates were relatively low with T_m_ at around 36 °C and 41 °C for D10 and G6, respectively (Fig. 3B). To further quantify target binding properties, kinetic analyses were performed using SPR. Monomeric D10 and G6 were injected over immobilized recombinant human CD40L, and binding responses were analyzed using a 1:1 Langmuir interaction model. Both candidates displayed binding affinities in the submicromolar range, with equilibrium dissociation constants (K_D_) of approximately 400 nM for D10 and 770 nM for G6 (Fig. 3C). The interaction profiles were characterized by relatively rapid association and dissociation kinetics. The specificity of D10 and G6 was assessed by flow cytometry analysis of *E. coli*-displayed clones against biotinylated hCD40L and three unrelated human proteins, hPD-1, hCD161, and h4-1BB. Both clones showed strong binding to hCD40L, while only background-level signals were observed for the unrelated proteins, supporting selective target recognition by the engineered affibody binders (Figure S5, Supplementary).

### Trimerization of affibody molecules using collagen domains

**Fig. 4:**
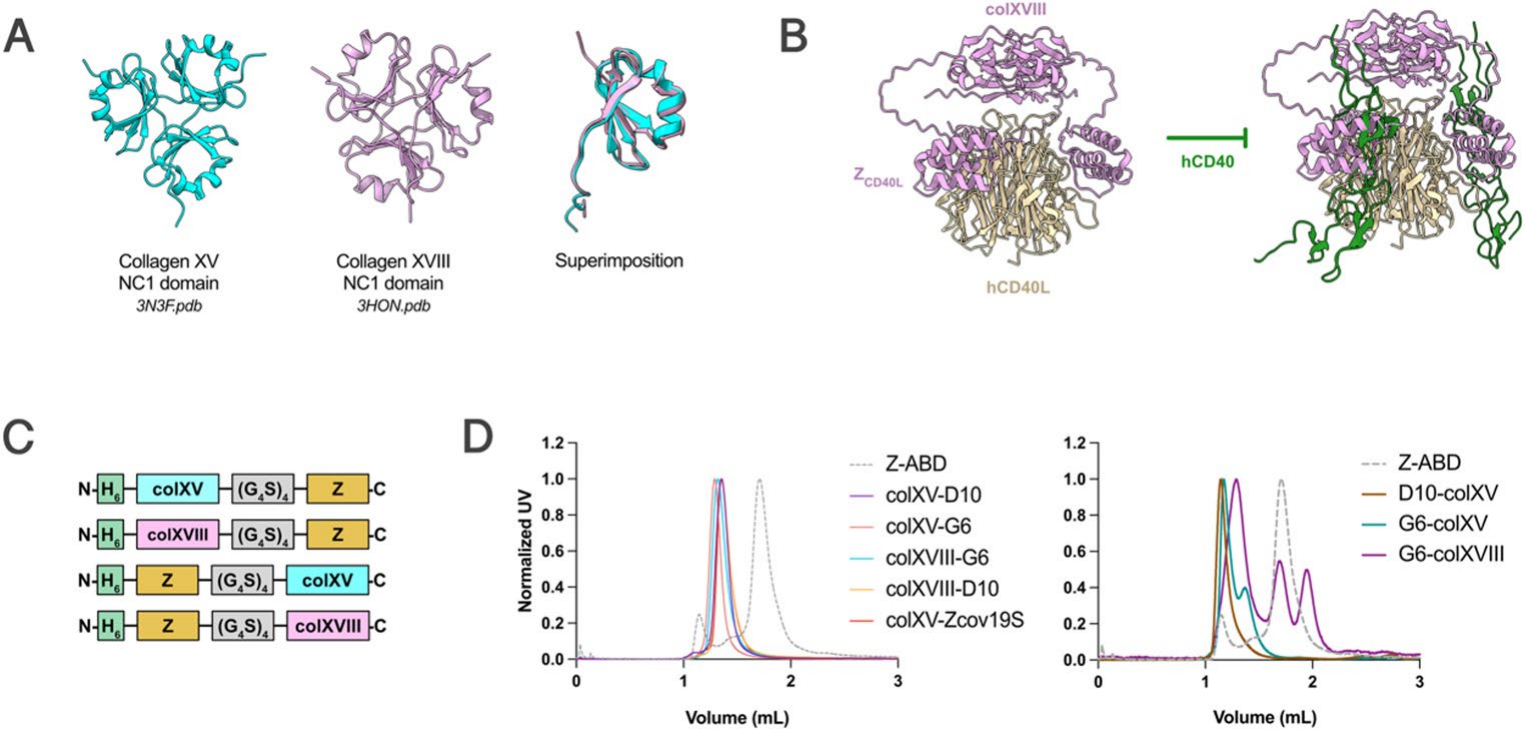
**(A)** Crystal structures of the NC1 domain from human collagen XV (3N3f.pdb) [25] and human collagen XVIII (3HON.pdb) [27]. Their respective structures are highly similar, as illustrated by the superimposition of their monomers to the right. **(B)** Suggested interaction geometry of hCD40L (beige) and the fusion protein candidate colXVIII-D10 (pink), superimposed with a co-crystal structure of human CD40 (green) and CD40L (3QD6.pdb) [31] to the right. **(C)** Construct design for fusion proteins with NC1 domains. H_6_ denotes a hexahistidine tag, Z an affibody molecule, (G_4_S)_4_ the glycine-serine spacer, and colXV and colXVIII the respective NC1 domains. **(D)** Chromatograms from analytical SEC of indicated fusion protein candidates or controls harboring the NC1 domain in the N-terminal (left) and C-terminal (right). Z-ABD was used as an arbitrary monomer of similar molecular size to a monomeric fusion protein candidate

To enhance the apparent affinity for human CD40L, the two affibody candidates were reformatted as trimeric constructs by genetic fusion to the NC1 domains of human collagen XV or collagen XVIII (Fig. 4A). Given that CD40L naturally assembles into homotrimers, we hypothesized that NC1-mediated trimerization of the affibody molecules could promote avidity-driven binding and thereby increase the apparent affinity. In addition, we reasoned that a trimeric affibody format could adopt a cap-like binding geometry on the CD40L trimer (Fig. 4B), potentially occluding CD40 interaction sites through steric hindrance.

The CD40L-binding affibody candidates D10 and G6 were reformatted together with two negative control affibodies, Z_wt_ [29] and Affi-A [30], referred to as Z_cov19S_, which bind IgG and the SARS-CoV-2 spike protein, respectively. For each affibody, four fusion protein architectures were generated in which the NC1 domain of collagen XV or collagen XVIII was positioned either N-terminally or C-terminally relative to the affibody domain, separated by a flexible (G₄S)₄ linker (Fig. 4C). The resulting constructs are hereafter referred to as colXV–Z, colXVIII–Z, Z–colXV, and Z–colXVIII, where Z denotes the affibody domain (D10 or G6). All fusion constructs were expressed recombinantly in *E. coli* and purified by IMAC. Purified proteins displayed the expected molecular sizes in SDS–PAGE analysis and all candidates apart from D10-colXVIII, which produced poorly, were obtained in sufficient purity and quantity for subsequent biophysical and functional characterization (Figure S6, Supplementary).

### Characterization of trimeric candidates

**Fig. 5:**
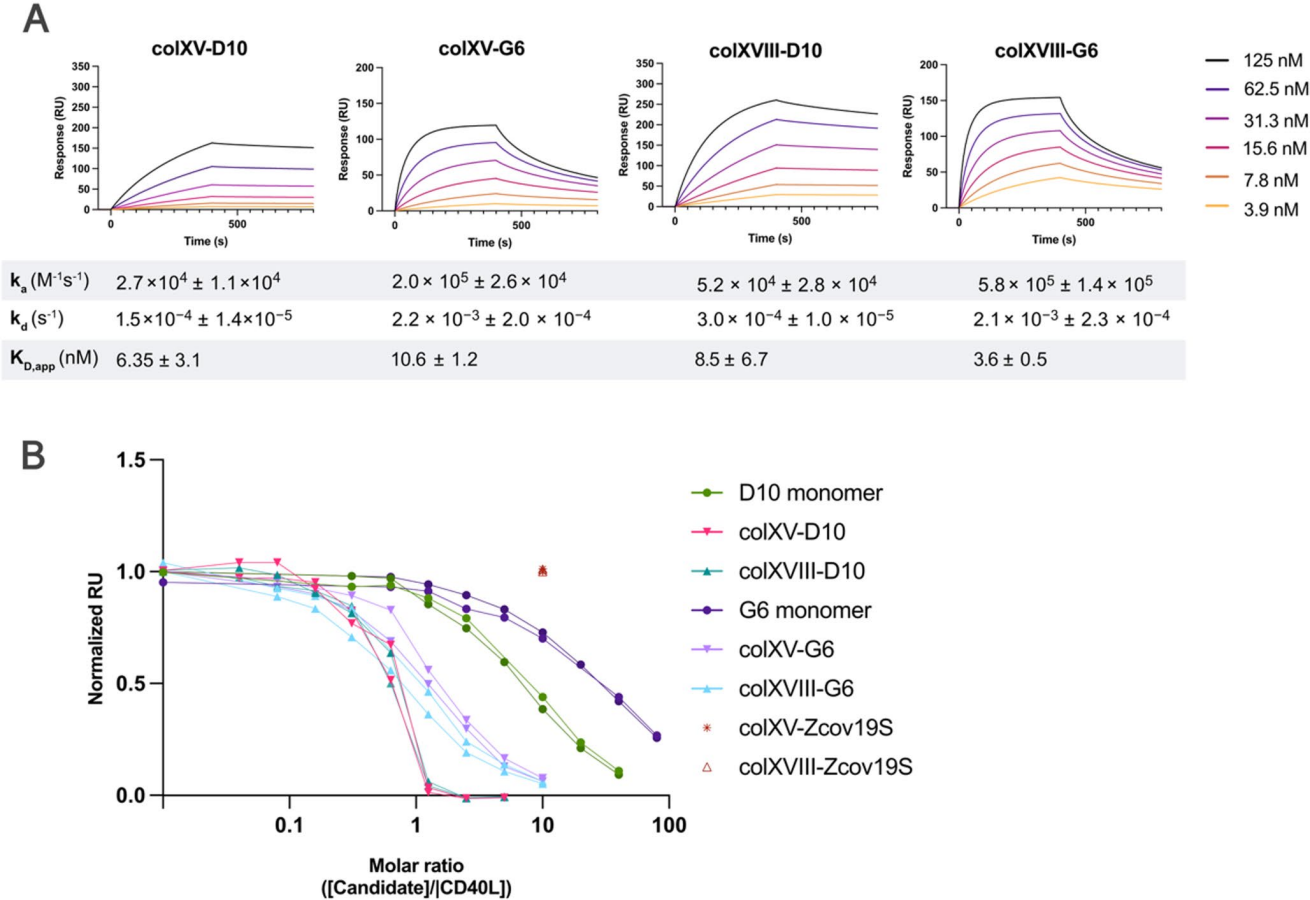
**(A)** Sensorgrams from the kinetic measurements of the N-terminal fusion protein candidates. The apparent kinetic constants were generated using a 1:1 Langmuir curve fitting model and are shown in the table. **(B)** Sensorgrams from two independent measurements of the CD40 blocking SPR assay. CD40 was immobilized on the chip surface and hCD40L, pre-incubated with the respective candidates or controls, was injected over the surface. The y-axis shows the normalized response units (RU) at equilibrium for each tested concentration of candidate, and the x-axis shows the molar ratio of candidate to CD40L

The oligomeric state of the affibody fusion constructs was evaluated by analytical size-exclusion chromatography (SEC). Constructs containing the collagen XV or collagen XVIII NC1 domain at the N terminus of the affibody (colXV–Z and colXVIII–Z) eluted as single, symmetric peaks, indicative of homogeneous species (Fig. 4D). The observed elution volumes corresponded well to the expected molecular size of functional homotrimers, approximately 66 kDa, when compared with the elution profile of the low-molecular-weight calibration standard (Figure S7, Supplementary). In contrast, constructs in which the collagen domains were positioned at the C terminus of the affibody (Z–colXV and Z–colXVIII) displayed broader and, in some cases, multiple elution peaks. This heterogeneity was particularly pronounced for G6–colXV and G6–colXVIII (Fig. 4D), suggesting incomplete or unstable trimer formation in this configuration. Based on these observations, all subsequent experiments were performed exclusively using constructs with N-terminal collagen domains (sequences are shown in Table S1, Supplementary). The thermal stability and refoldability of the trimeric candidates were assessed by CD spectroscopy. Fusion to the collagen NC1 domains did not result in a marked change in melting temperatures compared with the corresponding monomeric affibody candidates. However, differences in refoldability were observed following thermal denaturation. For the D10-based trimers, fusion to either collagen XV or collagen XVIII negatively affected refolding, whereas refoldability remained largely intact for the corresponding G6-based trimeric constructs (Figure S8, Supplementary). Binding kinetics of the trimeric candidates were next evaluated by SPR using recombinant trimeric human CD40L immobilized on the sensor surface. Compared with their monomeric counterparts, the trimeric constructs displayed pronounced avidity effects, most notably reflected in substantially reduced dissociation rates, supporting successful trimerization of the candidates (Fig. 5A). Association rates were also reduced for the trimeric formats, consistent with multivalent binding behavior. Overall, the apparent affinities (K_D, app_) of the trimeric candidates were in the low nanomolar range, corresponding to an approximately 100-fold improvement relative to the monomeric affibody candidates. The functional ability of the trimeric constructs to inhibit the CD40–CD40L interaction was subsequently evaluated using an SPR-based blocking assay. Monomeric and trimeric affibody candidates, along with matched trimeric negative controls (colXVIII–Z_cov19S_ and colXV–Z_cov19S_), were pre-incubated with recombinant human CD40L and injected over a sensor surface immobilized with recombinant human CD40. Importantly, both monomeric candidates, D10 and G6, displayed inhibition of CD40 binding at higher molar ratios relative to CD40L, indicating intrinsic antagonistic activity of the affibody molecules in monovalent format (Fig. 5B). Notably, NC1-mediated trimerization resulted in a pronounced enhancement of inhibitory efficacy. The trimeric constructs achieved substantially stronger CD40 blockade than their monomeric counterparts, corresponding to an approximate one- to two-order-of-magnitude improvement in functional blocking potency. Importantly, matched trimeric negative control constructs showed no detectable inhibition, confirming that the observed effects were specific to CD40L targeting.

### Interaction of trimeric affibody candidates with membrane-bound CD40L

**Fig. 6:**
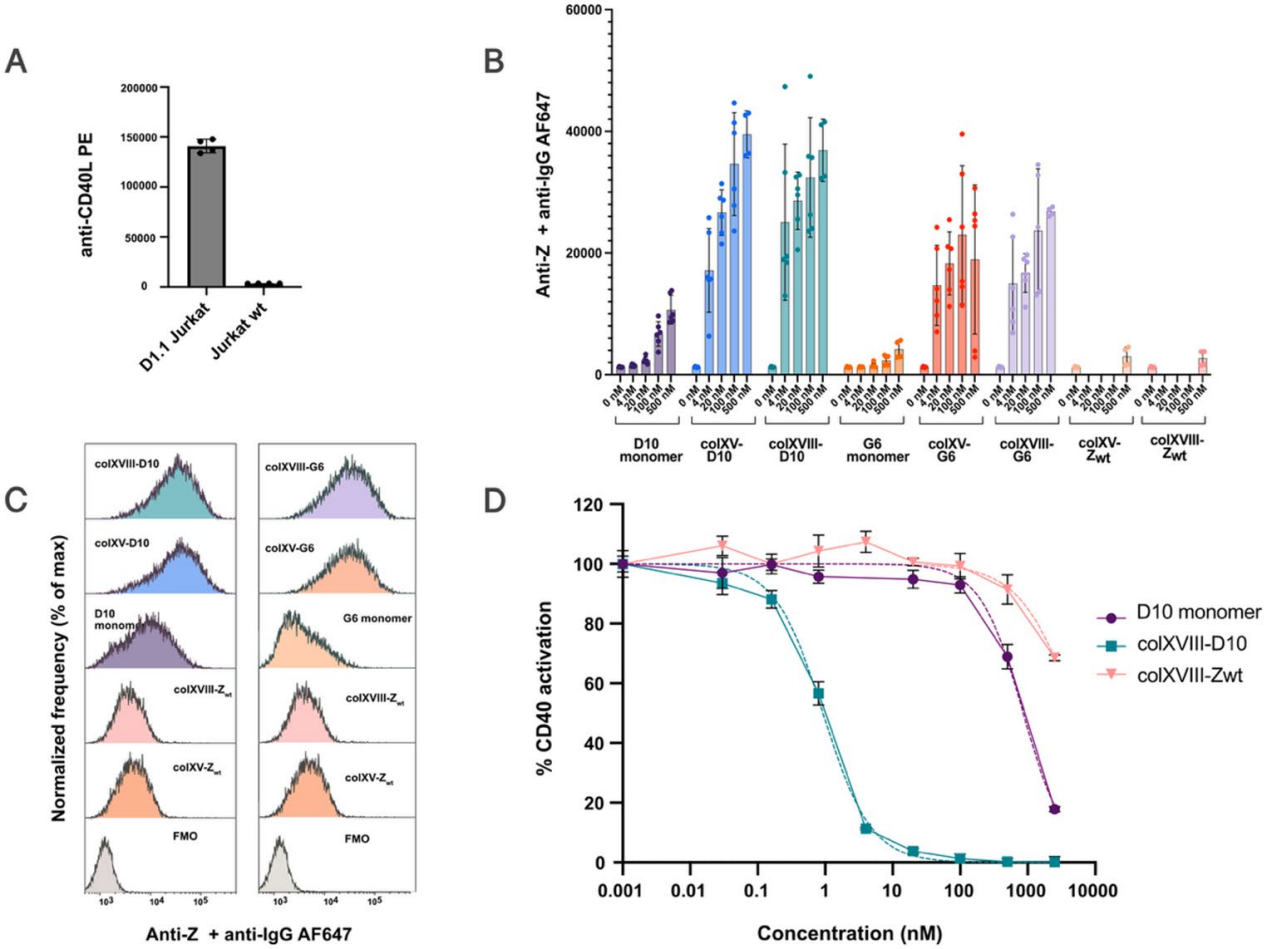
**(A)** Surface expression of CD40L, represented by the median fluorescent intensity (MFI), on Jurkat D1.1 cells and Jurkat wild-type (wt) cells following staining with a fluorescently labeled CD40L-specific monoclonal antibody. The data points represent duplicate values from two independent measurements. **(B)** Jurkat D1.1 cell binding (MFI) of monomeric and trimeric candidates or negative controls following labeling with the indicated concentrations. The data points represent duplicate values from three independent measurements. **(C)** Overlay of histograms displaying fluorescence intensity normalized to each population’s maximum signal (% of max) for the indicated candidates and negative controls at 100 nM, measured using the 638/655–665 nm laser/filter combination. **(D)** Inhibition curves from the NF-κB reporter assay showing CD40 blockade by monomeric D10 and colXVIII-D10, with colXVIII-Zwt included as a negative control

To evaluate whether the trimeric affibody candidates retained the ability to bind CD40L in its native, membrane-bound context, flow cytometry analyses were performed using mammalian cells constitutively expressing CD40L. Jurkat D1.1 cells, a subclone of the Jurkat lymphoblastoid T cell line with stable surface expression of CD40L [32], were used as a positive model system, while wild-type Jurkat cells lacking CD40L expression served as a negative control. A CD40L-specific monoclonal antibody was included to verify CD40L expression on the cell surface (Fig. 6A). Both monomeric and trimeric affibody candidates bound CD40L-expressing D1.1 cells in a concentration-dependent manner (Fig. 6B–C). In contrast, binding to CD40L-negative Jurkat wild-type cells was negligible across all concentrations tested (Figure S9, Supplementary), confirming CD40L-specific recognition. Strikingly, the trimeric affibody constructs showed a pronounced enhancement in apparent binding to cell-surface CD40L compared to their monomeric counterparts. This effect was particularly pronounced at low ligand concentrations. At the lowest tested concentration (4 nM), the trimeric candidates generated higher fluorescence signals than the corresponding monomeric affibody candidates at the highest tested concentration (500 nM), indicating a substantial avidity-driven gain in functional binding on the cell surface. Together, these results demonstrate that trimerization of CD40L-binding affibody molecules confers a marked increase in apparent cell-surface binding. Importantly, the enhanced binding was achieved without compromising target specificity.

### NF-κB reporter cell assay for CD40 inhibition

To determine whether the enhanced cell-surface binding observed for the trimeric affibody format translated into functional inhibition of CD40/CD40L signaling, a cell-based NF-κB reporter assay was employed. HEK-Blue CD40L reporter cells, which express CD40 and produce secreted embryonic alkaline phosphatase (SEAP) in response to NF-κB activation, were used to quantify pathway activation upon CD40L stimulation. The trimeric construct colXVIII-D10 was selected as the lead candidate for functional evaluation. Recombinant CD40L was pre-incubated with serial dilutions of either monomeric or trimeric D10 affibody candidates prior to stimulation of the reporter cells, enabling direct assessment of ligand neutralization. NF-κB activation was subsequently quantified by measuring SEAP activity in the culture supernatant following 20 h of stimulation. Stimulation with CD40L alone resulted in robust NF-κB activation, whereas no signal was detected in the absence of CD40L or upon stimulation with irrelevant cytokine controls, confirming assay specificity and low background activity. Pre-incubation of CD40L with the monomeric D10 affibody resulted in only weak inhibition of NF-κB signaling, which was detectable at high affibody concentrations. Dose–response analysis yielded an IC50 value of 1.0 ± 0.2 µM for the monomeric candidate. The trimeric D10 construct exhibited a pronounced and concentration-dependent inhibition of NF-κB activation. Inhibition was observed already at low nanomolar concentrations, with near-complete suppression of signaling at higher concentrations. Quantitative analysis of the dose–response curve revealed an IC50 value of 0.9 ± 0.34 nM, corresponding to an approximately three orders of magnitude improvement in functional inhibitory potency compared to the monomeric format (Fig. 6D). Importantly, no NF-κB activation or inhibition was observed when reporter cells were incubated with affibody candidates in the absence of CD40L, demonstrating that the observed effects were dependent on CD40L neutralization rather than nonspecific modulation of CD40 signaling. Together, these results demonstrate that trimerization of the CD40L-binding affibody not only enhances apparent binding through avidity effects but also translates into potent functional inhibition of CD40/CD40L-mediated NF-κB signaling.

## Discussion

CD40L is an attractive therapeutic target in autoimmune and inflammatory diseases due to its central role in CD40-mediated co-stimulation of adaptive immune responses. Previous clinical efforts to antagonize CD40L using conventional IgG antibodies were hampered by severe thromboembolic adverse events, attributed to Fc-mediated crosslinking of CD40L on platelets [17]. These safety concerns have motivated the exploration of alternative targeting strategies that avoid Fc-dependent effector functions, including engineered affinity proteins devoid of Fc domains [1]. In this study, we explored affibody molecules as Fc-free antagonists of CD40L. Using directed evolution in an *E. coli* surface display system, we isolated multiple CD40L-binding affibody candidates from a large naive library. Although the selection strategy yielded binders capable of recognizing CD40L both in recombinant and cellular contexts, biophysical characterization revealed that the monomeric affibody candidates exhibited relatively weak affinities in solution. This discrepancy between strong apparent binding during display-based selections and modest affinities in soluble format highlights a known limitation of high-valency systems like the *E. coli* display method used here, particularly when applied to multimeric targets such as CD40L [33]. Rather than pursuing conventional affinity maturation of the monomeric binders at this stage, we explored a format-based strategy to harness avidity through controlled multimerization. By genetically fusing affibody molecules to trimerization domains, we sought to match the native homotrimeric architecture of CD40L to potentially enhance functional engagement. To our knowledge, this is the first study to evaluate trimerization motifs derived from the NC1 domains of human collagen XV and XVIII as oligomerization modules for affibody molecules. These domains were selected due to their well-defined trimeric structure, human origin, and compact size, making them attractive candidates for multimerization of small affinity proteins [21]. Biophysical characterization using analytical size exclusion chromatography demonstrated that N-terminal fusion of the NC1 domains yielded homogeneous monodisperse elution profiles, in line with homotrimeric affibody assembly, whereas C-terminal placement resulted in heterogeneous assemblies, underscoring the importance of fusion geometry for reliable trimer formation. The resulting trimeric affibody constructs displayed pronounced avidity effects, most notably reflected in substantially reduced dissociation rates in SPR experiments and an approximately 100-fold improvement in apparent affinity relative to monomeric affibodies. Importantly, the avidity-driven affinity enhancement translated into improved biological function. Trimeric affibody constructs exhibited markedly enhanced binding to membrane-bound CD40L on Jurkat D1.1 cells and achieved strong cell-surface engagement at nanomolar concentrations where monomeric counterparts showed minimal binding. Notably, this improvement in target engagement was further reflected in functional assays, where the trimeric lead candidate potently inhibited CD40L-induced NF-κB activation in a reporter cell assay with an IC_50_ in the low nanomolar range, representing an approximately three orders of magnitude improvement in functional potency compared to the monomeric format. While these results establish NC1-mediated trimerization as an effective strategy for enhancing affibody functionality, some challenges remain. Both lead candidates exhibited relatively low thermal stability, which may limit downstream therapeutic development and highlights the need for further scaffold optimization. In this context, an attractive future strategy would be to combine classical affinity maturation of the monovalent affibody scaffold with subsequent NC1-mediated trimerization. Given the strong avidity effects observed here, such a sequential optimization approach has the potential to generate exceptionally potent CD40L antagonists by synergistically improving intrinsic affinity and multivalent target engagement.

In conclusion, this work demonstrates that trimerization via human collagen-derived NC1 domains represents a novel and effective strategy for enhancing the functional performance of affibody molecules against homotrimeric targets. By rationally matching binder valency with target architecture, this approach enables substantial gains in apparent affinity, cell-surface engagement, and functional inhibition without reliance on Fc-mediated mechanisms. Beyond CD40L, NC1-mediated trimerization may provide a generalizable platform for engineering multivalent affibody-based antagonists targeting other homotrimeric members of the TNF superfamily, thereby expanding the toolbox for next-generation, Fc-free affinity protein therapeutics.

## Supplementary Information

Below is the link to the electronic supplementary material.

Supplementary Material 1 (PDF 1.90 MB)

## Data Availability

The data underlying this article are available in the article and in its online supplementary material.
