# Correction: Trimerization of CD40L-specific affibody molecules using collagen domains enhances target binding and CD40 blockade

**DOI:** 10.1007/s00018-026-06384-x

**Published:** 2026-08-10

**Authors:** Cornelia Westerberg, Chiara Sorini, Mariam Al-Haddad, Hanna Mehari, Charlotta Preger, Stefan Ståhl, Maja Jagodic, John Löfblom

**Affiliations:** 1 Department of Protein Science, School of Engineering Sciences in Chemistry, Biotechnology and Health, KTH Royal Institute of Technology, Stockholm, 106 91 Sweden; 2 Department of Clinical Neuroscience, Center for Molecular Medicine, Karolinska Institutet, Karolinska University Hospital, Stockholm, 171 76 Sweden

**Correction: Cellular and Molecular Life Sciences (2026) 83:262**

**https://doi.org/10.1007/s00018-026-06301-2**

In this article, in the author order the author “Charlotta Preger” was missing, the correct order should have been read as “Cornelia Westerberg1 · Chiara Sorini2 · Mariam Al-Haddad1 · Hanna Mehari1 · Charlotta Preger1· Stefan Ståhl1 · Maja Jagodic2 · John Löfblom1”.

The original article has been initiated.

