# Supplementary material for: Trimerization of CD40L-specific affibody molecules using collagen domains enhances target binding and CD40 blockade
Source: Cell Mol Life Sci. 2026 Jun 24;83(1):262. doi: 10.1007/s00018-026-06301-2 (PMC13309602; doi:10.1007/s00018-026-06301-2)
Supplement: Supplementary file 1 — Supplementary Material 1 (PDF 1.90 MB) [file 18_2026_6301_MOESM1_ESM.pdf]

Supplementary material

Trimerization of CD40L-specific affibody molecules using collagen domains enhances target binding and CD40 blockade

Cornelia Westerberg[1], Chiara Sorini[2], Mariam Al-Haddad[1], Hanna Mehari[1], Stefan Ståhl[1], Maja Jagodic[2], John Löfblom[1*]

[1]Department of Protein Science, School of Engineering Sciences in Chemistry, Biotechnology and Health, KTH Royal Institute of Technology, 106 91 Stockholm, Sweden.

[2]Department of Clinical Neuroscience, Karolinska Institutet, Center for Molecular Medicine, Karolinska University Hospital, 171 76 Stockholm, Sweden.

[*]**Correspondence:** John Löfblom lofblom@kth.se

Table S1. Amino acid sequences of the NC1 subdomains from human collagen XV and XIII, respectively, used for trimerization.

Construct	Amino acid sequence
**Human collagen XV NC1**	NLVTAFSNMDDMLQKAHLVIEGTFIYLRDSTEFFIRVRDGWKKLQLGELIPIPA
**Human collagen XVIII NC1**	GSSGVRLWATRQAMLGQVHEVPEGWLIFVAEQEELYVRVQNGFRKVQLEAR TPLPR
**colXV-D10**	GSSHHHHHHNLVTAFSNMDDMLQKAHLVIEGTFIYLRDSTEFFIRVRDGWKK LQLGELIPIPAGGGGSGGGGSGGGGSGGGGSYYLEVDNKFWKEWTKAWDEIY YLPNLNTSQRLAFDKSLSDDPSQSANLLAEAKKLNDAQAPK
**colXVIII-D10**	GSSHHHHHHGSSGVRLWATRQAMLGQVHEVPEGWLIFVAEQEELYVRVQNG FRKVQLEARTPLPRGGGGSGGGGSGGGGSGGGGSYYLEVDNKFNKETSDAL WEITYLPNLNIKQRWAFRDSLHDDPSQSANLLAEAKKLNDAQAPK
**colXV-G6**	GSSHHHHHHNLVTAFSNMDDMLQKAHLVIEGTFIYLRDSTEFFIRVRDGWKK LQLGELIPIPAGGGGSGGGGSGGGGSGGGGSYYLEVDNKFNKEVHTAHREIEY LPNLNEVQKDAFIYSLLDDPSQSANLLAEAKKLNDAQAPK
**colXVIII-G6**	GSSHHHHHHGSSGVRLWATRQAMLGQVHEVPEGWLIFVAEQEELYVRVQNG FRKVQLEARTPLPRGGGGSGGGGSGGGGSGGGGSYYLEVDNKFNKEVHTAH REIEYLPNLNEVQKDAFIYSLLDDPSQSANLLAEAKKLNDAQAPK

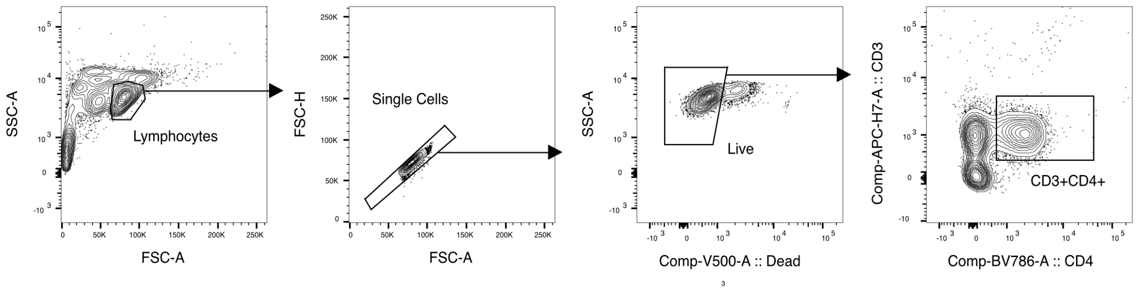

Figure S1. Gating strategy used for the identification of $CD3^{+}CD4^{+}$ T lymphocytes during the assessment of affibody binding to human primary T cells.

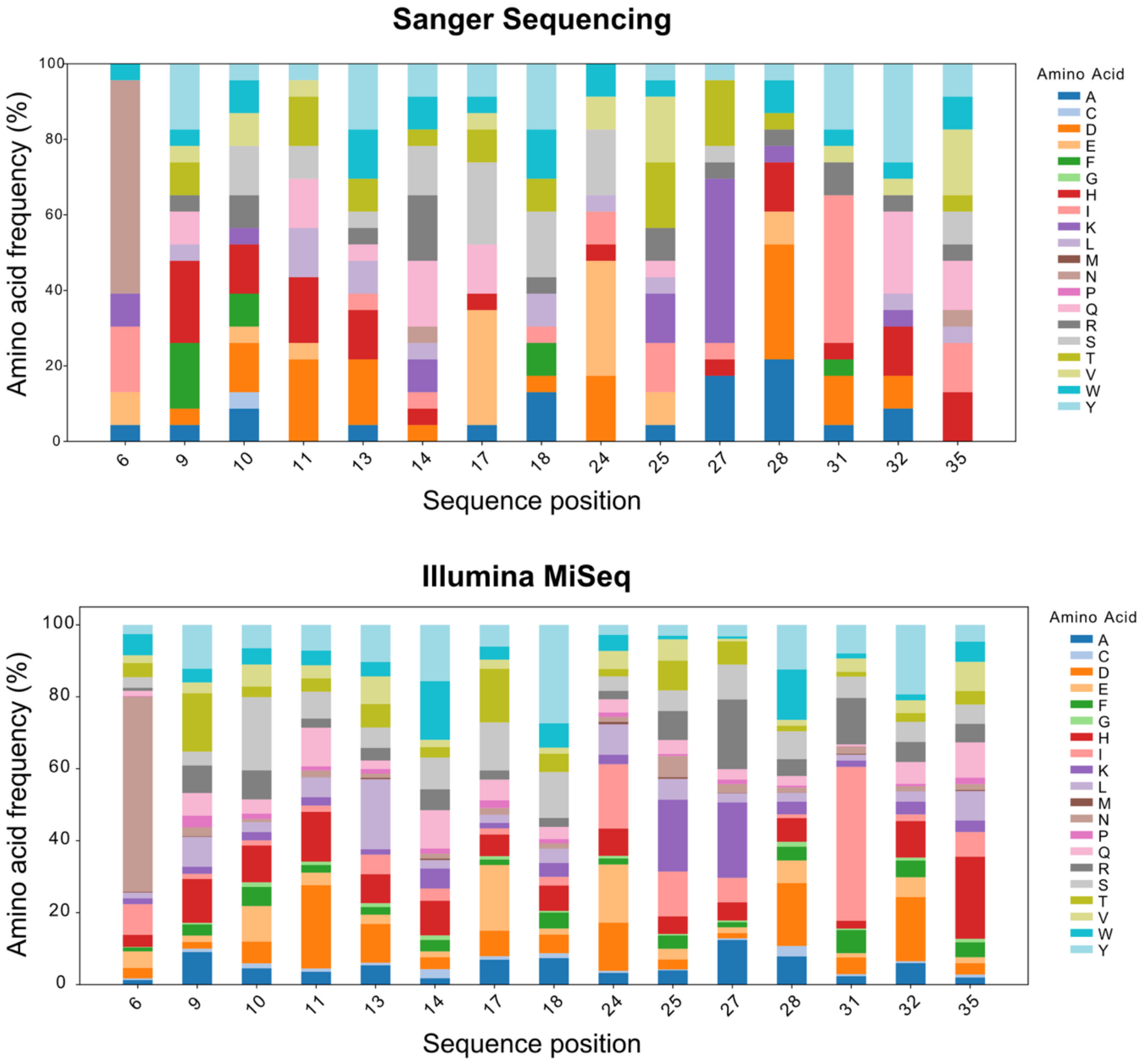

Figure S2. Amino acid bar charts for Sanger sequencing data (top) and Illumina MiSeq data (bottom). The respective frequencies of amino acids are shown for the indicated diversified positions in the affibody molecule.

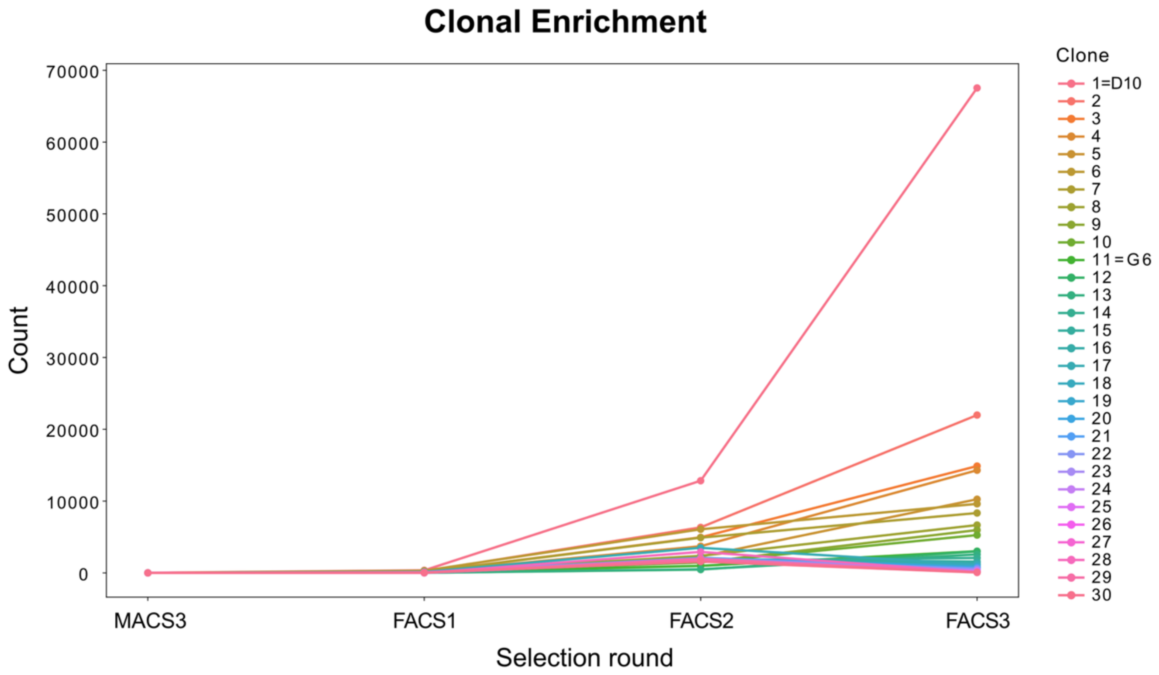

Figure S3. Enrichment trajectories from MACS 3 to FACS 3 for the thirty most enriched clones. The most enriched clone corresponds to D10, while G6 was the eleventh most enriched in a set of 32 500 unique clones.

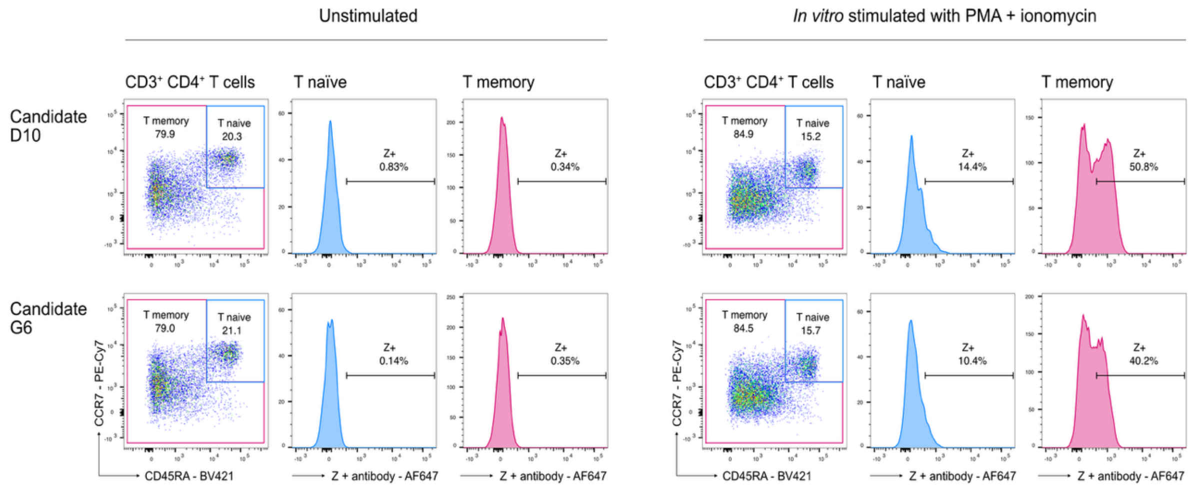

Figure S4. Flow cytometry analysis showing binding of candidate affibodies to CD3$^+$CD4$^+$ T cells from human peripheral blood mononuclear cells (PBMCs) without stimulation (left) and upon *in vitro* stimulation with phorbol myristate acetate (PMA) and ionomycin (right).

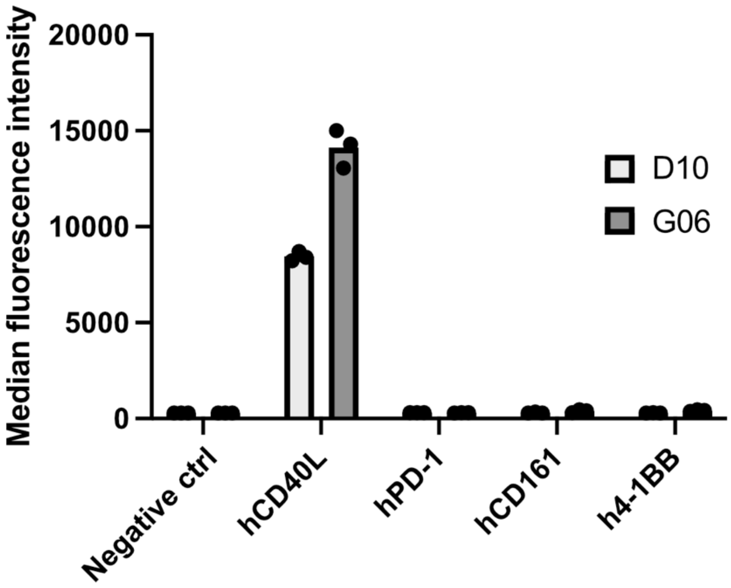

Figure S5. Flow cytometry analysis of binding specificity for the *E. coli*-displayed affibody variants D10 and G06. Binding to 100 nM biotinylated human CD40L (hCD40L) and three unrelated proteins, human PD-1 (hPD-1), human CD161 (hCD161), and human 4-1BB (h4-1BB), was evaluated. Bound target proteins were detected using streptavidin–phycoerythrin (SAPE). The negative control consisted of SAPE only. Median fluorescence intensity is shown as mean values from three replicates, with individual data points indicated.

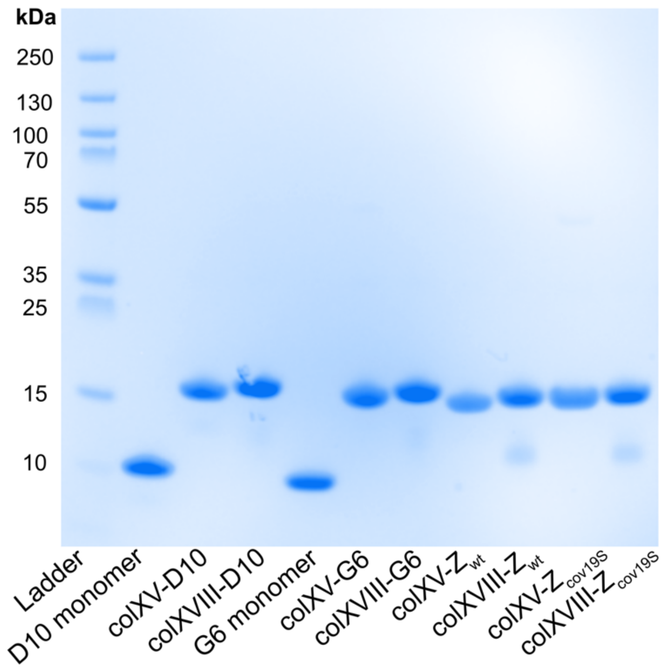

Figure S6. SDS-PAGE gel showing the purified monomeric and trimeric candidates and negative controls.

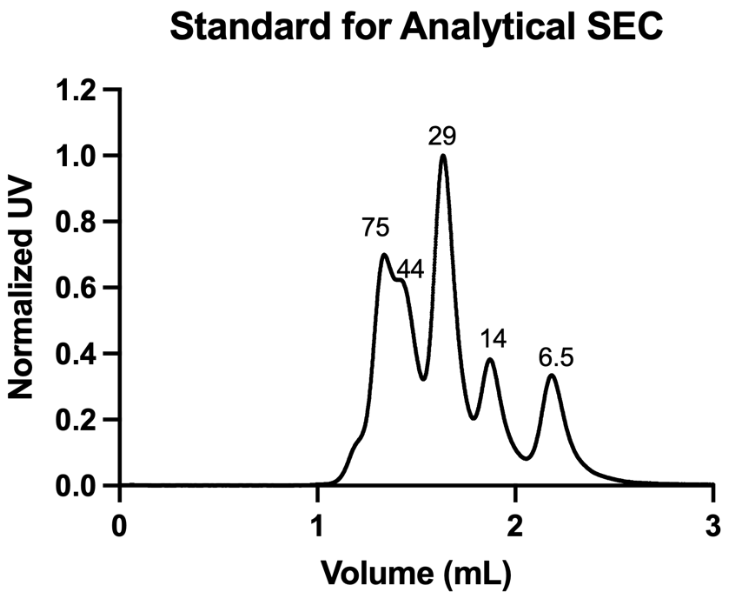

Figure S7. Chromatogram showing the elution peaks for the standard used when running analytical SEC on the trimeric candidates. Molecular sizes (in kDa) of the standard proteins are indicated above each elution peak.

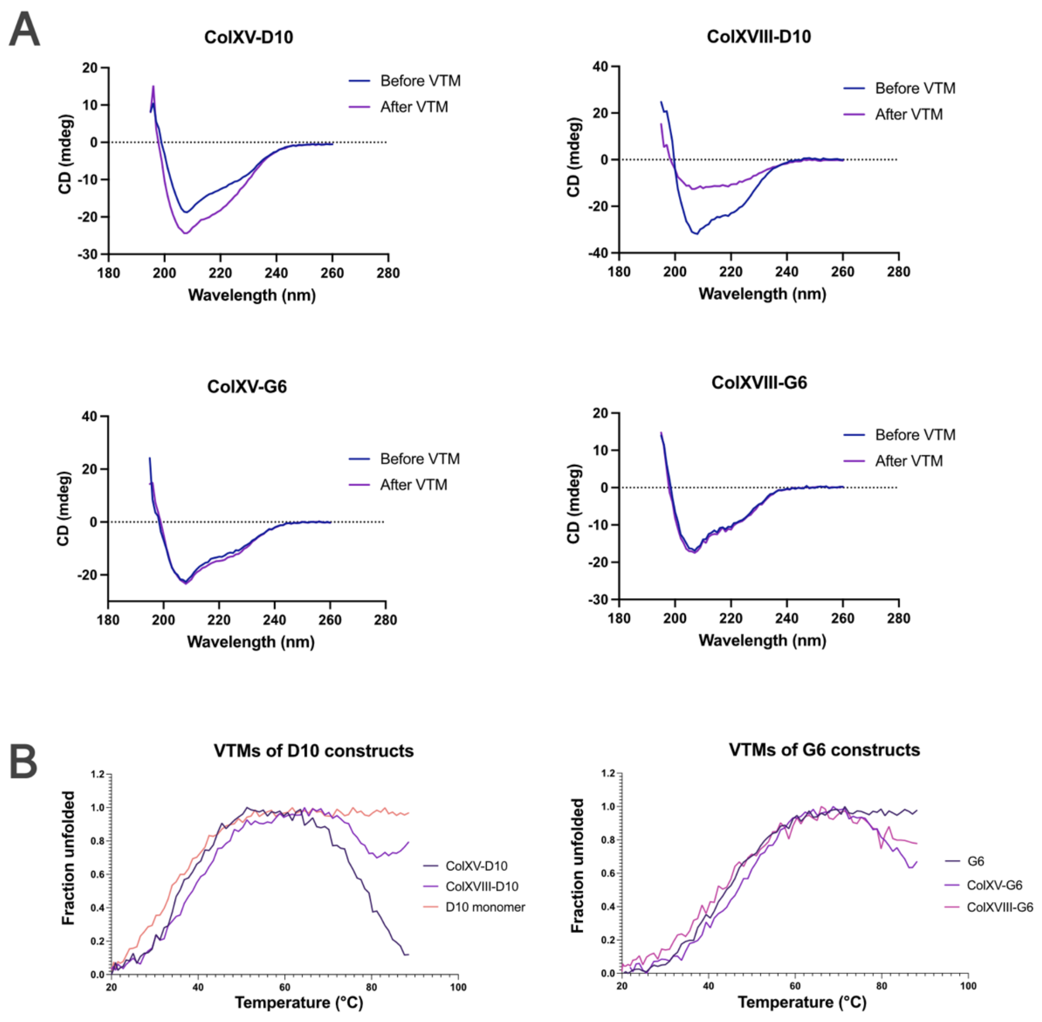

Figure S8. A) CD spectra before and after heat denaturation and **B)** variable temperature measurements (VTMs) for the trimeric candidates.

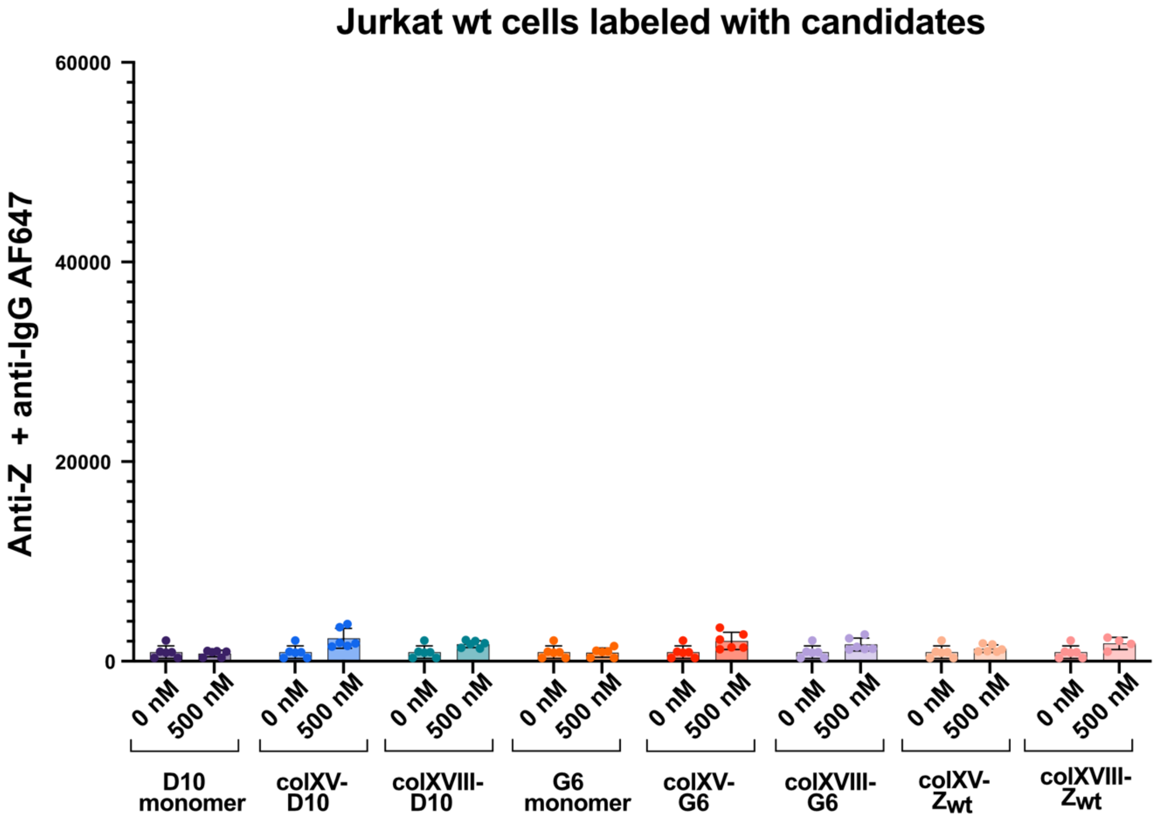

Figure S9. Jurkat wild-type binding (MFI) of monomeric and trimeric candidates or negative controls following labeling with the indicated concentrations. The data points represent duplicate values from three independent measurements.
